# Mutation patterns in colorectal cancer and their relationship with prognosis

**DOI:** 10.1016/j.heliyon.2024.e36550

**Published:** 2024-08-20

**Authors:** Zhaoran Su, Maria El Hage, Michael Linnebacher

**Affiliations:** a Department of Gastrointestinal Surgery, People's Hospital of Tongling City, China; b College of Mathematics and Computer Science, Tongling University, Tongling 244000, China; c Molecular Oncology and Immunotherapy, Clinic of General Surgery, University Medical Center Rostock, Rostock 18057, Germany

**Keywords:** Colorectal cancer, Mutation, Biomarker, Prognosis

## Abstract

**Background:**

Colorectal cancer (CRC) is a prevalent malignancy and a leading cause of cancer-related mortality. Extensive research into the aetiology of CRC has revealed that somatic mutations in certain genes play a crucial role in CRC development.

AIM: In this study, we utilized data from public databases to investigate prevalent mutation patterns in CRC and developed a prognostic predictive model for CRC patients based on mutant genetic characteristics and other relevant clinical features.

**Methods:**

We initially gathered mutation information from CRC patients by analysing data from 15 datasets to identify genes with a mutation frequency of ≥10 %. Next, log-rank analyses were used to determine the relationship between prognosis and the mutational status of the most commonly mutated genes; the SIGnaling database was utilized to generate a protein‒protein interaction network. We consolidated and classified the gene mutation patterns of CRC patients in the database based on frequently mutated genes related to prognosis. A predictive nomogram was constructed, including age, sex, TNM stage, and mutation partner, based on available clinical, mutational, and prognostic information for CRC patients at our institution. Finally, the reliability of the model was verified using time-dependent ROC curve analysis.

**Results:**

The top 7 genes somatically mutated ≥10 % in 4477 samples from 4255 patients were *TP53* (67 %), *APC* (66 %), *KRAS* (43 %), *PIK3CA* (18 %), *FBXW7* (14 %), *SMAD4* (14 %), and *BRAF* (10 %). Log-rank analysis demonstrated that the mutation status of 5 genes, namely, *TP53*, *APC*, *PIK3CA*, *SMAD4*, and *BRAF*, correlated significantly with prognosis. Protein‒protein interaction analysis confirmed functional interactions between these 5 genes, implicating them in tumorigenesis. We exhaustively enumerated the mutation patterns involving these five genes in 4255 patients, resulting in identification of 32 mutational patterns. After consolidation and classification, these patterns were divided into 3 grades based on patient prognosis. Next, a predictive nomogram based on the clinical, mutational, and prognostic information of 107 CRC patients treated at University Medical Center Rostock was constructed. The area under the curve (AUC) values for the model for predicting 1-, 3-, and 5-year overall survival were 0.779, 0.721, and 0.815, respectively.

**Conclusion:**

Common mutational patterns based on frequently mutated genes are associated with prognosis in CRC patients. Our study provides a valuable and concise prognostic predictor for determining outcomes in patients with CRC.

## ARTICLE Highlights

• Seven genes with mutation rates ≥10 % were identified in CRC: TP53, APC, KRAS, PIK3CA, FBXW7, SMAD4, and BRAF.
• Mutations in TP53, APC, PIK3CA, SMAD4, and BRAF significantly affect prognosis. 32 mutational patterns of these genes were classified into three prognostic grades.
• A predictive nomogram integrating clinical, mutational, and prognostic data demonstrated strong predictive accuracy.
• The findings provide clinicians a tool for assessing CRC outcomes and guiding treatment, potentially improving patient care.

## 1 Introduction

Colorectal cancer (CRC) is a highly prevalent malignancy that remains a leading cause of cancer-related mortality [1]. Extensive research into the aetiology of CRC has revealed that its development involves multiple stages and the cumulative effects of various factors. Abnormal activation of molecular signalling pathways and genes due to mutations play a significant role in the formation and progression of CRC [2,3]. Specifically, certain mutations in tumour-suppressor genes have been identified as crucial biological triggers for CRC. In fact, more than 80 % of CRC patients carry common mutations in multiple tumour-suppressor genes [[4], [5], [6]]. Despite decades of research, the interrelationships among these mutations and their combined impact on prognosis are not yet fully understood.

Advances in genetic sequencing technologies have revolutionized the field. They enable broad and rapid assessment of numerous genes and samples, leading to improved diagnosis and prognosis and potential prediction of treatment response for CRC patients [7,8]. Nonetheless, identifying multivariate molecular factors that allow for the most accurate prediction of prognosis remains a major challenge. Therefore, efforts to decipher the complex relationships between different molecular alterations and their collective influence on the course of CRC are mandatory.

In this study, we used data from public databases to analyse prevalent mutation patterns in CRC. Using machine learning, we then assessed the potential influence of these patterns on patient survival. Additionally, we verified the relationship between the obtained mutation model and patient prognosis using real-world data from our clinic. Finally, we developed a robust predictive model for CRC patients based on somatic genetic characteristics while taking other relevant clinical features into account. The primary focus was overall survival (OS) as an outcome measure. This predictive model will assist clinically in providing more effective and personalized care for individuals with CRC.

## 2 Materials and Methods

### 2.1 Mutation data collection and analysis from public databases

We initially gathered somatic mutation information from CRC patients by analysing data from 15 datasets [[9], [10], [11], [12], [13], [14], [15], [16], [17], [18], [19], [20], [21]]: appendiceal cancer (MSK, J Clin Oncol 2022), colon adenocarcinoma (CaseCCC, PNAS 2015), colon cancer (CPTAC-2 Prospective, Cell 2019), colorectal adenocarcinoma (DFCI, Cell Reports 2016), colorectal adenocarcinoma (Genentech, Nature 2012), colorectal adenocarcinoma (MSK, Nat Commun 2022), colorectal adenocarcinoma (TCGA, Firehose Legacy), colorectal adenocarcinoma triplets (MSK, Genome Biol 2014), colorectal cancer (MSK, Cancer Discovery 2022), colorectal cancer (MSK, Gastroenterology 2020), colorectal cancer (MSK, JCO Precis Oncol 2022), disparities in metastatic colorectal cancer between Africans and Americans (MSK, 2020), metastatic colorectal cancer (MSK, Cancer Cell 2018), rectal cancer (MSK, Nature Medicine 2022) and rectal cancer (MSK, Nature Medicine 2019). The most frequently mutated genes in CRC samples were assessed across the 15 datasets, and genes with a mutation frequency of at least 10 % were identified as highly relevant. To ensure robust data processing, we established a key criterion in our study: the detection frequency of potential frequently mutated genes must be at least 80 % of the total sample size. This criterion helps eliminate potential batch effects during data analysis, thereby ensuring the reliability of results. cBioPortal (https://www.cbioportal.org/) is a web-based bioinformatics platform which can integrate and visualize complex genomic data from various cancer studies, providing an interface to explore gene mutations, copy number alterations, and clinical data; it was utilized to visualize the mutational landscape of the top mutated genes. Through utilization of cBioPortal and the GenVisR package (version 4.0.3), we analysed the correlations between mutation data and clinical phenotypes and pathological features from 15 datasets, and exhaustively explored all combinatorial patterns of high-frequency mutated genes.

### 2.2 Machine learning for mutation patterns and prognosis

Valid OS information of patients from the 15 datasets was collected. First, log-rank analyses were used to study the relationship between the prognosis and mutation status of the most common mutated genes with frequencies greater than 10 %. Mutations with P values less than 0.100 were incorporated into the mutation pattern analysis. Based on the high-frequency mutated genes and related clinical outcome data of the CRC patients included in the 15 datasets, we tested all gene mutation combinations. Subsequently, by using clustering algorithms in machine learning, the mutational patterns were gathered and classified according to OS. This allowed us to divide the mutational patterns into different groups, with cases within the same group showing high similarity and cases between different groups showing less similar outcome. An agglomerative clustering algorithm, a machine learning library based on the python programming language (Python 3.9.0, Python Software Foundation), was used for unsupervised learning to cluster the mutation patterns based on the OS of patients. The parameter settings were as follows: number of clusters (n_clusters): 3, linkage criterion (linkage): Ward's variance minimization criterion, similarity measurement method (affinity): Euclidean distance (“euclidean”). After clustering the mutation patterns based on OS using the agglomerative clustering algorithm, we performed additional log-rank tests to evaluate the effectiveness of the clustering. This comparison allowed us to examine whether there were significant differences in OS between the different clusters and to verify if the clusters effectively represented distinct prognostic groups. The log-rank test assessed whether the survival distributions of the patients in different clusters were statistically different, thereby validating the success of the clustering in separating patients into groups with different prognoses.

### 2.3 Analysis of interactions or functional relationships between biomolecules

To investigate the relationship between prognosis-related high-frequency mutated genes and tumorigenesis, we used SIGnaling Network Open Resource (SIGNOR 3.0), which is, available online at http://signor.uniroma2.it. By inputting the identified high-frequency mutated genes into the SIGNOR platform, an interactive network was constructed to visualize and explore the functional interactions between these genes within different signaling pathways. This network analysis allowed us to investigate whether these mutated genes interact with each other and how these interactions may collectively contribute to tumorigenesis. A confidence score >0.5 was used as a cut-off criterion.

### 2.4 Clinical sample and information collection

To confirm the results obtained from public databases, we analysed data from patients at Rostock University Medical Center. We collected 125 CRC tissues from 107 CRC patients who underwent surgical treatment at the Clinic of General Surgery between January 2006 and December 2019. Tissue and data collection for research purposes was approved by the Ethics Committee of the University Medical Center Rostock (II HV 43/2004, A45/2007, A2018-0054, and A2019-0187). All patients signed informed consent forms. There were 56 males and 51 females among the 107 patients, with an average age of 69.2 years. According to the 7th edition of the UICC staging system, there were 9 patients with stage I disease, 26 with stage II disease, 28 with stage III disease and 44 with stage IV disease. The detailed characteristics of the patients are listed in Table 1 and Supplementary material 1.

**Table 1:** The characteristics of the 107 HROC patients.

Age (years)	69.2 ± 12.6
Sex (n,%)	
male	56 (52.3)
female	51 (47.7)
TNM stage (n,%)	
I	9 (8.4)
II	26 (24.2)
III	28 (26.1)
IV	44 (41.1)
Molecular subtype^a^(n,%)	
CIN	66 (52.8)
spMSI-H	29 (23.2)
Lynch Syndrome	10 (8.0)
CIMP-H	9 (7.2)
CIMP-L	10 (8.0)
Neuroendocrine	1 (0.8)
Adjuvant treatment (n,%)	
with	58 (54.2)
without	49 (45.8)

a A total of 125 samples were obtained from the primary tumours and metastases of 107 patients.

### 2.5 Patient-derived xenograft (PDX) models and genetic mutation detection

To preserve the genetic and phenotypic diversity of patient tumour tissues, tumour models were established as patient-derived xenograft (PDX) mouse models for these 125 tumours. Somatic mutations in the models were subsequently determined. This research resource has been described in detail previously [22]. Whole-exome sequencing (WES) analyses were conducted on 20 patient-derived xenografts (PDXs) following genomic DNA extraction. The remaining analyses utilized Centogene's solid tumour panel, which included 105 fully sequenced genes and mutational hot spots from an additional 146 genes. The technical details of the sequencing procedure were also previously described [23]. The Twist Library Preparation Enzymatic Fragmentation Kit (Twist Bioscience, San Francisco, CA, USA) was used for library preparation. Exome enrichment was performed using either the TWIST Human Core Exome Plus probes, covering 36.5 Mb of the human coding exome, or custom probes for the Solid Tumor panel. Sequencing was performed on the NextSeq 500 (for the Solid Tumor panel) or HiSeq 4000 and NovaSeq (for WES) systems from Illumina, Inc. (San Diego, CA, USA), generating 2 × 150 bp reads. Raw sequencing reads were converted to standard fastq format using Illumina's bcl2fastq software 2.17.1.14. The short reads were then aligned to the human reference genome GRCh37 (hg19) using Bowtie version 2.4.2. After alignment, samtools v. 1.11 was used for sorting, and PicardTools v. 2.23.8 was used for deduplication. Variant calling was done using the Strelka Somatic pipeline (v. 2.9.2). The resulting variant table was filtered using vcftools v. 0.1.16 and annotated with snpEff. The filters applied included protein-coding mutations, the “pass” filter, allele frequency >5 %, quality >50, and a minimum of 20 reads for the tumour. Only pathogenic or likely pathogenic mutations and mutations of uncertain significance were included; benign, risk factor- and drug response-influencing mutations were excluded. Additionally, only mutations from the raw data that met the following quality criteria are listed: passed the “Pass” filter, were not coding synonyms, had a quality score ≥30, and had a variant allele frequency of at least 15 %. If different samples from the same patient showed different mutation statuses for the genes of interest, the overall mutation information for that patient was determined using the union approach.

### 2.6 Construction of the prognostic model and verification

All 107 CRC patients were routinely followed up, with the last update in December 2020. To identify final independent prognostic biomarkers, we used multivariate Cox regression analysis to analyse associations between OS of the 107 patients and potential prognostic factors such as age, sex, TNM stage, molecular subtype, receipt of adjuvant treatment combined with the grades of mutation patterns. Using the backward stepwise selection method of Cox analysis, we identified independent prognostic factors by adjusting for other variables iteratively removing variables with the highest p-values until only statistically significant factors (p < 0.05) remained. Based on the final set of independent risk factors identified by multivariate Cox regression analysis, we constructed a predictive nomogram to estimate individual patient survival probabilities at 1, 3, and 5 years. The nomogram was developed using the rms package in R. Each prognostic factor is assigned a score based on its relative contribution to the model, and the total score is used to predict survival probabilities. Receiver operating characteristic (ROC) curves were generated to evaluate the performance of the predictive nomogram for 1-, 3-, and 5-year survival. For each time point, the ROC curve was constructed by plotting the true positive rate (sensitivity) against the false positive rate (1 - specificity), which provided a graphical representation of nomogram's ability to distinguish between different outcome states. The area under the curve (AUC) values are used to evaluate the performance of predictions.

### 2.7 Statistical analysis

All statistical analyses were performed with the SPSS 19.0 and R 4.0.3 software programs. Log-rank analyses were used to study the relationship between prognosis and the genes with frequent mutations in the 15 datasets. If the P value was less than 0.100, the gene was included in the mutation pattern analysis. The machine learning-based clustering algorithm was implemented by using Python 3.9.0 software and related program packages. Survival curves were analysed by using the Kaplan–Meier method. Multivariate Cox regression analyses were used to study independent risk factors for OS. A P value < 0.05 was considered to indicate statistical significance. ROC curves were generated to evaluate the performance of the predictive nomogram for predicting 1-, 3-, and 5-year survival.

## 3 Results

### 3.1 Gene mutation frequency and commonly mutated genes in CRC

**Fig. 1:**
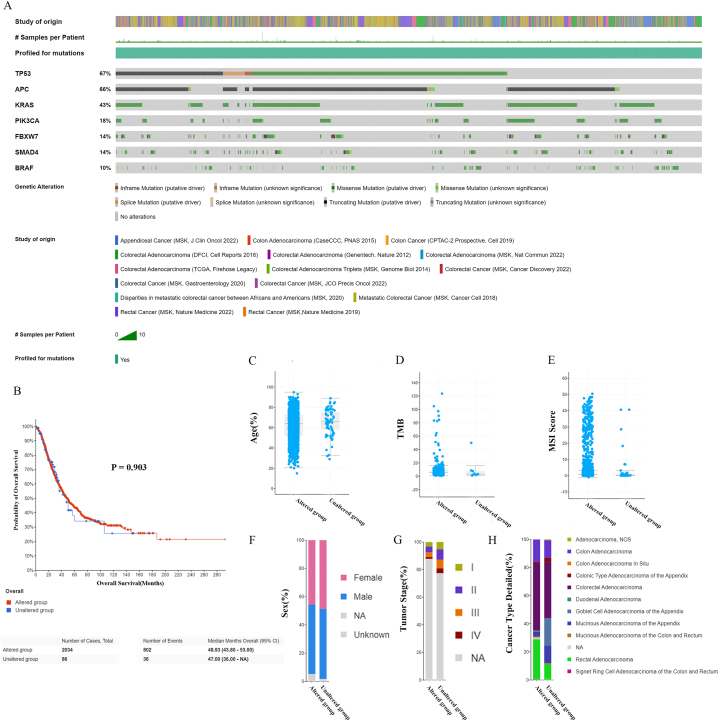
Mutational landscape of CRC.The top seven frequently mutated genes with mutations ≥10 % in 4477 samples from 4255 patients according to analysis of 15 datasets are shown in a waterfall plot (A). Available clinical and pathological data of the two groups with (altered group) or without (unaltered group) genetic mutations in at least one of the seven genes, namely, overall survival (B), age (C), TMB (D), MSI score (E), sex (F), tumour stage (G), and cancer type (H), are shown.

Our initial analysis revealed that the top seven mutated genes, with mutation rates ≥10 % in 4477 samples of 4255 patients from the 15 CRC datasets included, were *TP53* (67 %), *APC* (66 %), *KRAS* (43 %), *PIK3CA* (18 %), *FBXW7* (14 %), *SMAD4* (14 %) and *BRAF* (10 %). The landscape of the top 7 gene mutation profiles is shown in Fig. 1A. At least one of the related gene mutations was detected in 4302 samples from 4083 patients (95.9 %). However, no mutations in any of the aforementioned genes were detected in 175 samples from 172 (4.0 %) patients. The available clinical and pathological data of the two groups of patients included OS (Fig. 1B), age (Fig. 1C), tumour mutational burden (Fig. 1D), microsatellite instability score (Fig. 1E), sex (Fig. 1F), tumour stage (Fig. 1G), and cancer subtype (Fig. 1H).

### 3.2 Gene mutations and prognosis

**Fig. 2:**
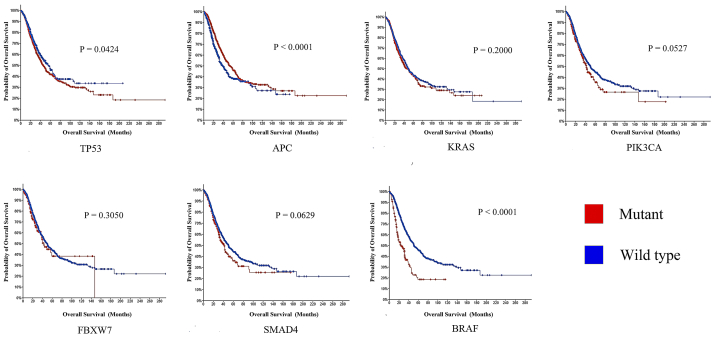
Prognostic ability of the top seven frequently mutated genes in predicting the OS of CRC patients. K‒M plots were used to analyse the difference in survival between the two groups with and without at least one somatic mutation, and the results revealed that *TP53*, *APC*, *PIK3CA*, *SMAD4* and *BRAF* correlated significantly with patient prognosis (P < 0.100).

Valid OS information was available for 2119 patients of the 15 datasets. Log-rank analyses were first used to study the relationship between prognosis and the mutational status of the seven most common mutated genes. These analyses revealed that mutations in five of the seven genes (*TP53*, *APC*, *PIK3CA*, *SMAD4* and *BRAF*) correlated significantly with patient prognosis (P < 0.100, Fig. 2).

### 3.3 Analysis of interactions or functional relationships between biomolecules

**Fig. 3:**
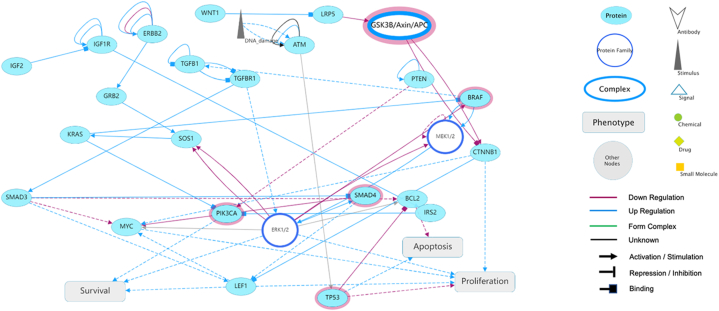
Biological informatics via SIGNOR.SIGNOR analysis concerning the relevance of different biological entities, namely, TP53, APC, PIK3CA, SMAD4 and BRAF, to CRC was performed. The obtained network contained 29 nodes and 61 edges with predicted participation in functional signal transduction as well as regulation of tumour cell survival, apoptosis, and proliferation.

The above five genes were selected to construct a protein‒protein interaction and signalling interaction network. In SIGNOR, these five genes are positioned with regard to biological entities first in human CRC, containing 29 nodes and 61 edges, and are predicted to participate in functional signal transduction, regulation of tumour cell survival, apoptosis, and proliferation (similarity: 0.54, P < 0.010; Fig. 3).

### 3.4 Mutation patterns and prognosis

**Fig. 4:**
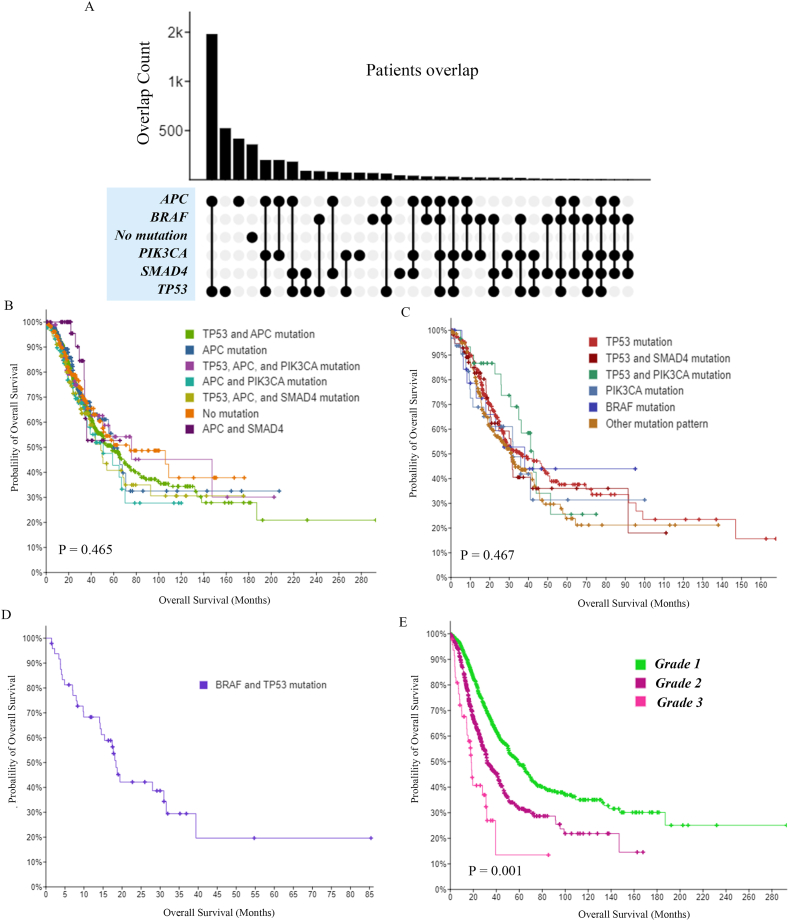
Machine learning for identifying mutation patterns and prognostic subgroups. Thirty-two single-gene and multiple-gene mutation patterns were found after exhaustively enumerating the distribution patterns of mutations involving the five preselected genes in 4255 patients. The number of patients (overlapping count) with each mutation pattern is indicated (black dots) (A). Based on patient OS, mutation patterns were classified into three groups by machine learning using the agglomerative clustering algorithm, there was no significant difference in prognosis among cases with different mutation patterns within each group. (B, C, D). The prognosis of the 3 patient groups differed significantly (E).

We thoroughly enumerated the distribution patterns of mutations involving the five genes in 4255 patients. The results revealed 32 mutation patterns including single-gene, multiple-gene and no mutation, as shown in Fig. 4A. We then used the median survival time to split mutation patterns into different prognostic types (Fig. 4B, C, D). After the data were merged, the patients were divided into 3 groups according to their prognosis: Group 1 had the best prognosis and Group 3 the worst prognosis, as shown in Table 2. There was no significant difference in the prognosis of patients with different mutations in their respective groups, but as expected, the prognosis of the 3 groups of patients was significantly different (Fig. 4E; P = 0.001).

**Table 2:** Analysis of mutation partner clustering based on prognosis.

Mutation partner	Number of Cases	Number of Events	Median Months Overall (95 % CI)
Group 1			
TP53 and APC mutation	720	265	58.33 (50.27–69.97)
APC mutation	191	59	59.93 (55.30 - NA)
TP53, APC, and PIK3CA mutation	94	30	75.80 (51.17 - NA)
APC and PIK3CA mutation	91	36	50.60 (35.57 - NA)
TP53, APC, and SMAD4 mutation	89	40	48.50 (38.70–92.77)
no mutation	215	66	74.00 (50.00 - NA)
APC and SMAD4	40	7	NA
total	1440	503	**58.63 (51.30**–**66.87)**
**Group 2**			
TP53 mutation	276	128	35.64 (27.90–49.93)
TP53 and SMAD4 mutation	56	26	31.07 (20.67 - NA)
TP53 and PIK3CA mutation	30	14	42.60 (35.00 - NA)
PIK3CA mutation	32	15	32.00 (17.00 - NA)
BRAF mutation	24	9	38.04 (18.00 - NA)
other mutation patterns	213	113	31.17 (24.73–41.87)
total	631	305	**32.00 (30.37**–**41.00)**
**Group 3**			
BRAF and TP53 mutation	48	29	**18.40 (14.53 - NA)**

### 3.5 Clinical sample collection and genetic mutation detection

**Fig. 5:**
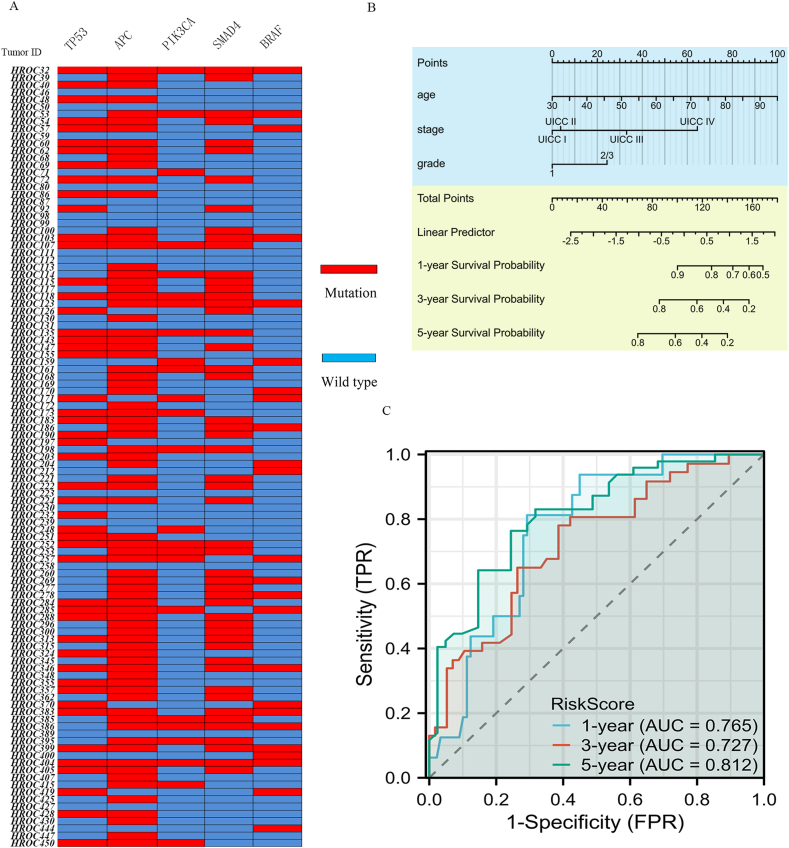
Construction of a prognostic nomogram for CRC patients. A detailed description of the mutation patterns of 107 patients from University Medical Center Rostock is displayed (A). The mutation rates of the five genes *TP53*, *APC*, *PIK3CA*, *SMAD4* and *BRAF* were 44.9 % (48/107), 71.9 % (77/107), 21.5 % (23/107), 46.7 % (50/107) and 21.5 % (23/107), respectively. Predicted 1-, 3-, and 5-year survival rates of these CRC patients based on the prognostic nomogram, which included age, mutation patterns, and UICC stage (B). The area under the curve (AUC) values for the model for predicting 1-, 3-, and 5-year OS were 0.765, 0.727, and 0.812, respectively (C). Points: The single scores correspond to each predicted variable. Total Points: sum of the single score points. Linear Predictor: weighted sum of the variables in the Cox regression model, with high values indicating worse prognosis.

We collected CRC tissues from 125 samples of 107 patients undergoing treatment at the Clinic of General Surgery, University Medical Center Rostock. After establishment of PDX mouse models, the somatic mutations preserved in all samples were identified by sequencing. Relevant clinical data were collected, and CRC molecular subtypes were determined (Supplementary material 1). The mutation rates of the five preselected genes *TP53*, *APC*, *PIK3CA*, *SMAD4* and *BRAF* were 44.9 % (48/107), 71.9 % (77/107), 21.5 % (23/107), 46.7 % (50/107), and 21.5 % (23/107), respectively. A detailed description of the mutation patterns is shown in Fig. 5A and Supplementary material 2.

### 3.6 Construction and evaluation of a nomogram

To identify independent prognostic biomarkers, we used univariate Cox regression analysis for associations between OS and the following factors: mutation pattern, age, sex, TNM stage, CRC molecular subtype and adjuvant treatment status. Age, TNM stage, and mutation pattern were found to be independent risk factors for OS (Table 3). We constructed a predictive nomogram based on these criteria as previously described (Fig. 5B). ROC analysis was performed, and the AUC values for the model for predicting 1-, 3-, and 5-year OS were 0.765, 0.727, and 0.812, respectively (Fig. 5C).

**Table 3:** Cox regression analysis for OS. UICC stage (P < 0.001), age (P = 0.001) and mutation pattern (P = 0.010) were found to be independent prognostic factors for OS.

Characteristics	n	Beta	HR	95 % CI	P value
Sex					
female	51		Reference		
male	56	0.50356	1.655	0.911-3.005	0.098
**Age**		0.05914	1.061	1.027-1.096	<**0.001**
**Stage**					
UICC IV	44		Reference		
UICC II	26	−1.7714	0.170	0.073-0.397	<**0.001**
UICC III	28	−1.0657	0.345	0.172-0.689	**0.003**
UICC I	9	−1.8961	0.150	0.034-0.665	**0.013**
**Mutation partner group**					
1	70		Reference		
2/3	37	0.82048	2.272	1.215-4.247	**0.010**
**Molecular subtype**					
spMSI-H	29		Reference		
CIN	48	−0.30434	0.738	0.370-1.469	0.387
neuroendocrine	1	−15.382	0.000	0.000 - Inf	0.996
CIMP-L	10	1.135	3.111	0.177-1.224	0.122
CIMP-H	9	−0.081574	0.922	0.319-2.666	0.880
Lynch Syndrome	10	0.015164	1.015	0.353-2.920	0.978
**Adjuvant treatment**					
with	58		Reference		
without	49	−0.17447	0.840	0.445-1.585	0.590

## 4 Discussion

In this study, data from public databases were utilized to identify the most prevalent mutation patterns in CRC. By using machine learning, the mutation patterns were then constrained into three levels according to the median survival time of patients through a classification algorithm. Then, a reliable and concise prognostic model was constructed based only on age, TNM stage, and mutation pattern. It showed satisfactory performance in predicting OS in an independent sample cohort of CRC patients.

According to the somatic mutations reported in the 15 public datasets included, the most commonly mutated genes in CRC are *TP53*, *APC*, *KRAS*, *PIK3CA*, *FBXW7*, *SMAD4*, and *BRAF*. Log-rank analysis revealed that among these seven genes, *KRAS* and *FBXW7* were not prognostically related, whereas the mutational status of the remaining five genes, *TP53*, *APC*, *PIK3CA*, *SMAD4*, and *BRAF,* correlated significantly with prognosis. We were able to confirm the results from the abovementioned public data with a cohort of CRC patients from our clinic. Based on the sequencing results of the latter, *TP53*, *APC*, *PIK3CA*, *SMAD4,* and *BRAF* exhibited similarly high somatic mutation frequencies of 44.9 %, 71.9 %, 21.5 %, 46.7 %, and 21.5 %, respectively.

Protein‒protein interaction analysis confirmed functional interactions between these 5 genes, implicating them in CRC tumorigenesis. Genetic mutations play a major role in CRC development [24]. *APC* is one of the most commonly mutated genes in CRC. Indeed, it is mutated in the germline of nearly all patients with familial adenomatous polyposis and in a majority of patients with nonfamilial CRC [25]. This mutation leads to abnormal activation of the Wnt signalling pathway within cells, thereby promoting tumour development [26]. *TP53* is considered a prototypical “cancer gene” that plays a critical role in maintaining the stability of the genome. *TP53* mutations are associated with malignant progression and poor prognosis in CRC patients [27,28]. The *BRAF*^V600E^ mutation is relatively common in CRC patients with high microsatellite instability. It has recently been associated with shorter OS [29]. The presence of somatic mutations in the *SMAD4* gene in CRC correlates significantly positively with the risk of distant metastasis [30]. However, *PIK3CA* mutations have only neutral prognostic effects on CRC overall survival (OS) and progression-free survival (PFS) [31]. K‒M analysis of the seven initially identified genes using publicly available data revealed that *TP53*, *APC*, *PIK3CA*, *SMAD4,* and *BRAF* correlated significantly with the prognosis of CRC patients, confirming previous results [[32], [33], [34]].

Surprisingly, in the initial analysis of 4302 patient samples, the impact of the five genes on prognosis was not unidirectional. The combination of somatic mutations in one tumour was found to impact prognosis. Thus, after identifying prognostically relevant high-frequency mutations using log-rank analysis, we subsequently tested combinations of mutations or mutation patterns. When enumerating the mutation patterns involving the five genes in the 4255 patients, prognostically relevant somatic mutation patterns could indeed be identified. The most common combinations of mutations in both the online databases and the data from our patients at University Medical Center in Rostock were comparable.

The finding that the somatic mutation pattern of a given CRC significantly influences patient prognosis is clearly of clinical relevance. For example, compared to patients without mutations in any of the five genes *TP53, APC, PIK3CA, SMAD4*, and *BRAF,* patients with a single *APC* gene mutation exhibited significantly worse outcomes (median months OS: *APC* mutation vs. no mutation, 59.93 vs. 74.00). However, compared to patients with only a *TP53* somatic mutation, patients with an *APC* mutation in addition to *TP53* mutation had better prognosis (median months OS: *TP53* and *APC* mutation vs. *TP53* mutation, 58.33 vs. 35.64). These findings are consistent with those of Schell and coworkers [35], who identified 17 genes involved in CRC by exome sequencing of 1321 cancer-related genes in 468 tumour specimens. *APC* mutations are central in predicting OS, and patients with tumours without *APC* mutation have worse prognosis than those with single somatic *APC* mutations. However, the combination of *APC*, *TP53* and *KRAS* confer the poorest survival among all the subgroups examined. Therefore, considering these data together with the results of the present study, it seems reasonable to conclude that accurate prognostic models must consider somatic mutation combinations rather than single individual genes with somatic mutations.

Then, we stratified patients into 3 groups according to survival prognosis: group one had the best prognosis, and group three had the worst prognosis. The three levels were differentiated according to the median survival time due to the mutational pattern of the five genes. This was achieved through a classification algorithm based on machine learning. In recent years, machine learning has been applied for in-depth analysis of large datasets. It has been implemented in the establishment of prognostic signatures, in radiomics, and in proteomics studies [[36], [37], [38]]. It is considered to be superior to cluster analysis for these applications. In the present study, the prognostic components of patients with three hierarchical mutation patterns clustered based on public databases varied widely but maintained good similarity within groups. Moreover, based on the results of univariate analysis, the most common combination of mutations in both the online databases and the data for our patients at University Medical Center in Rostock were similar, confirming the role of mutational patterns rather than single genes in affecting prognosis.

In the final step, we developed a simple but accurate monogram model to predict CRC patient survival. This model can be readily implemented in daily clinical decision-making processes. It is based on only 3 prognostic hub elements, including TNM stage, age, and somatic mutational patterns, as identified before. In addition, the compact model design allows for comparably easy validation at other institutions. We constructed a monogram model with the clinical, somatic mutation, and prognostic data for 107 CRC patients treated in our clinic and obtained similar AUC values for prediction of 1-, 3-, and 5-year OS (0.765, 0.727, and 0.812, respectively). This indicates that our monogram has good sensitivity and specificity for predicting both short-term and long-term outcomes, which in turn confirms its clinical relevance.

In this study, we focused on exploring the association between overall patterns of gene mutations and overall survival in CRC patients. However, we acknowledge that there are many other factors that may have biased the outcomes. Specifically, we did not assess the differential impact of mutation subtype on prognosis. Previous results have shown that subtypes may lead to different prognostic outcomes due to their distinctive biological features [39]. Therefore, future research should further explore specific mutation subtypes and their unique contributions to tumour initiation and progression.

## 5 Conclusion

Common patterns of somatic tumour mutations, which are based on the most frequently mutated genes, are significantly associated with the prognosis of CRC patients. The simple prognostic predictor developed in the present study by implementing the abovementioned observation may prove to be very useful for stratifying CRC patients into prognostic groups. This might be of high clinical relevance since it could easily be used in daily clinical practice as a guide for treatment decision-making.

## Funding

No funding was received.

## Institutional review board statement

Informed consent was obtained from all human subjects involved in the study. Tissue and data collection for research purposes was approved by the Ethics Committee of the University Medical Center Rostock (II HV 43/2004, A45/2007, A2018-0054, and A2019-0187).

## Clinical trial registration statement

Our investigation is solely observational, devoid of any interventions. Thus, it aligns with observational research rather than interventional research. Consequently, no clinical trial registration was pursued for this study.

## Informed consent statement

All study participants or their legal guardians provided informed written consent for personal and medical data collection prior to study enrolment.

## Data sharing statement

The original anonymous dataset is available upon request from the corresponding author at Michael.linnebacher@med.uni-rostock.de.

## CRediT authorship contribution statement

**Zhaoran Su:** Writing – review & editing, Writing – original draft, Investigation, Data curation, Conceptualization. **Maria El Hage:** Writing – review & editing, Validation, Data curation. **Michael Linnebacher:** Writing – review & editing, Supervision, Resources, Project administration, Conceptualization.

## Declaration of competing interest

The authors declare that they have no known competing financial interests or personal relationships that could have appeared to influence the work reported in this paper.
